# Special Issue “Molecular Research in Bone and Soft-Tissue Tumors”

**DOI:** 10.3390/ijms27209362

**Published:** 2026-10-21

**Authors:** Shinji Miwa, Katsuhiro Hayashi, Satoru Demura

**Affiliations:** Department of Orthopaedic Surgery, Graduate School of Medical Sciences, Kanazawa University, Kanazawa 920-8640, Japan

Although the developments in multidisciplinary treatment have significantly improved clinical outcomes in bone and soft-tissue sarcomas, the outcomes of patients with metastatic/recurrent sarcoma remain poor. Therefore, novel systemic treatments including molecular targeting agents and immunotherapy are in high demand to improve the clinical outcomes of patients with unresectable sarcomas. Recently, there has been a large number of studies on therapeutic targets and clinical trials for molecular targeting agents in bone and soft-tissue sarcomas. Furthermore, immune checkpoint inhibitors have shown superior outcomes compared to standard chemotherapy in several types of malignancies. This Special Issue aimed to bring together the latest research and reviews on therapeutic targets, immune microenvironment, molecular mechanisms, preclinical models, and novel treatments in bone and soft-tissue sarcomas.

In the treatment of chondrosarcoma, only tumor resection with appropriate surgical margin has been considered as curative treatment. On the other hand, clinical outcomes of patients with metastatic or unresectable chondrosarcoma remain unsatisfactory due to resistance to chemotherapy and radiation therapy. Landuzzi et al. summarize recent advances in the molecular biology, preclinical modeling, and therapeutic development of chondrosarcoma (Contribution 1). *IDH1/2* mutations, which occur in approximately 50–80% of conventional and dedifferentiated chondrosarcomas, induce widespread epigenetic alterations through the accumulation of the oncometabolite D-2-hydroxyglutarate. The authors discuss the advantages and disadvantages of conventional cell lines, three-dimensional spheroids, organoids, chick chorion-allantoic membrane models, genetically engineered mouse models, and patient-derived xenografts (PDX). Among these in preclinical modeling, PDX and PDX-derived cell lines are thought to be vital platforms for advancing drug discovery, because these models faithfully replicate the genetic alterations of tumors, especially in high-risk and treatment-resistant subtypes. This review also provides an extensive overview of emerging therapeutic approaches including mutant IDH inhibitors, the targeting of the SIRT-1-HIF-2α/NAD+ metabolic pathway, DR5 agonists such as INBRX-109, anti-angiogenic agents including pazopanib and regorafenib, epigenetic therapies, and immunotherapeutic approaches. Importantly, clinical studies of DR-5-targeted therapy and IDH inhibitors have demonstrated encouraging disease-control rates suggesting that these treatments can represent potential treatment options in patients with advanced chondrosarcomas.

Chordomas are rare malignant bone tumors, originating from remnants of notochordal cells. The transcription factor brachyury, encoded by *TBXT*, is essential for tumor growth. Although our understanding of chordoma biology has advanced, there are no approved targeted therapies. Seeling et al. investigated the effects of cytoskeletal inhibitors, including the actin-targeting compounds cucurbitacin B (CuB) and latrunculin B (LatB) on three chordoma cell lines (Contribution 2). Both CuB and LatB induced significant morphological changes in tumor cells, but only CuB caused significant depletion of brachyury. Microarray analysis demonstrated that treatment with CuB upregulated protein refolding and downregulated protein glycosylation. In contrast, CuB treatment slightly suppressed *TBXT* transcription, indicating that brachyury depletion was post-transcriptional. Furthermore, rescue experiments and co-treatments implicated dysregulated protein refolding and endoplasmic reticulum stress as key mechanisms underlying CuB-mediated brachyury depletion. These findings suggest that actin cytoskeleton dynamics or protein unfolding pathways are promising therapeutic targets for chordoma.

Malignant peripheral nerve sheath tumors (MPNSTs) are the most common malignant tumors in patients with neurofibromatosis type 1 but can also occur sporadically. Because most MPNSTs depend on signaling pathways associated with oxidative stress, therapeutic strategies that further potentiate oxidative stress may selectively eradicate tumor cells at doses that are tolerable for normal cells. Artemisinin derivatives have antimalarial effects due to an endoperoxide bridge in its chemical structure that induces oxidative stress. Duensing et al. reported that artesunate (ARS) and metabolite dihydroartemisinin (DHA) showed selective cytotoxic effects on MPNST cells relative to normal cells (Contribution 3). DHA induced oxidative stress and lipid peroxidation, whereas DHA-mediated cytotoxicity was inhibited with co-administration of the antioxidant N-acetyl-cysteine. Furthermore, MPNST was selectively inhibited by DHA in co-culture with normal Schwann cells. These findings suggest that artemisinins are a promising treatment for patients with MPNST.

Pleomorphic rhabdomyosarcoma (pRMS) is a rare and aggressive subtype of rhabdomyosarcoma, with both pleomorphic and skeletal muscle features. Because of the rarity, aggressiveness, and lack of detailed characterization, therapeutic treatment remains challenging. Kopantseva et al. performed single-cell transcriptomics to investigate the heterogeneous landscape and the tumor microenvironment in pRMS (Contribution 4). In this study, the tumor populations in pRMS could be classified into myogenic and non-myogenic clusters, and non-myogenic clusters had more numerous communication links with the immune populations. The immune response was suppressed by the MIF-CD74 pathway in all pRMS clusters and APP, PTN, and CXCL12 signaling in the non-myogenic tumor clusters. The cytotoxic T cells in pRMS showed exhaustion markers (LAG3, HAVCR2, EOMES), and the macrophages expressed myeloid checkpoint-related genes (*SIGLEC1*, *SIRPA*, *CSF1R*, *HAVCR2*). These transcriptomic findings suggest that MIF and APP signaling may be therapeutic target in pRMS.

Barr reviewed the diversity of molecular changes and clinical significance of gene amplifications in alveolar rhabdomyosarcoma (Contribution 5). Alveolar rhabdomyosarcomas have diverse patterns of gene amplification, involving protein coding genes such as *MYCN*, *CDK4*, and *SHMT2*, as well as the non-coding RNA (MIR17HG). These amplifications can occur through multistep processes, including translocation that forms a gene fusion (*PAX7::FOXO1*) followed by amplification. Furthermore, preclinical studies revealed that some amplification events (*CDK4* and *SHMT2*) reduce sensitivity to drugs targeting the amplified gene products while these amplifications increase sensitivity to agents targeting downstream of these amplified gene products. These studies emphasized the biological and clinical complexities of gene amplification.

The studies and reviews in this Special Issue focused on the tumor microenvironment, molecular heterogeneity, therapeutic targets, preclinical models in bone and soft-tissue sarcomas. Advances in single-cell analysis and preclinical modeling may enable the identification of promising therapeutic approaches in the treatment of advanced sarcomas. Although translation of these molecular studies into clinical application remains challenging, the studies and reviews in this Special Issue highlight the potential of molecular discoveries to contribute to improving clinical outcomes. We sincerely thank all the authors who contributed their valuable studies and reviews to this Special Issue. We hope that the collected articles will stimulate further research and contribute to the development of novel therapeutic strategies for bone and soft-tissue sarcomas.

## Data Availability

No new data were created or analyzed in this study. Data sharing is not applicable to this article.

